# Pharmacy practice and policy research in Türkiye: a systematic review of literature

**DOI:** 10.1080/20523211.2024.2385939

**Published:** 2024-08-12

**Authors:** Gizem Gülpınar, Aysel Pehlivanlı, Zaheer Ud-Din Babaar

**Affiliations:** a Department of Pharmacy Management, Faculty of Pharmacy, Gazi University, Ankara, Türkiye; b Department of Pharmacology, Faculty of Pharmacy, Baskent University, Ankara, Türkiye; c Clinical Pharmacy and Drug Information Center, Baskent University Ankara Hospital, Ankara, Türkiye; d Medicines and Healthcare, Department of Pharmacy, University of Huddersfield, Huddersfield, UK

**Keywords:** Pharmacy services, health policy, prescribing, medicine access, pharmacovigilance

## Abstract

**Background:**

In recent decades, there has been an interest in clinical pharmacy practice in Türkiye with emerging studies in this area. Despite the recent emergence of diverse pharmacy practice studies in Türkiye, a comprehensive assessment of overall typology of studies and impact has not been conducted thus far.

**Objectives:**

This systematic review aims to document and assess pharmaceutical policy and practice literature published within the last 5 years in Türkiye. The other aim is to summarise the expected impact of published studies on policy and practice research.

**Methods:**

The systematic review was conducted according to the guidelines described in the PRISMA Statement. A comprehensive search approach, incorporating Medical Subject Headings (MeSH) queries and free-text terms was employed to locate pertinent literature related to pharmacy practice and policy in Türkiye. The search covered the period from January 1, 2019, to January 1, 2024, and involved electronic databases including PubMed, Medline Ovid, Scopus, ScienceDirect, Springer Link, PlosOne, and BMC.

**Results:**

In the final grouping, 73 articles met the inclusion criteria and were selected for this review. Among the quantitative studies, majority studies were cross-sectional survey studies. Through the rigorous thematic content analysis seven research domains were developed from the selected literature: drug utilisation and rational drug use, the emerging role of pharmacist, access to medicines and generic medicines, community pharmacy practice, pharmacovigilance/adverse drug reactions, and pharmacoeconomic studies.

**Conclusions:**

The pharmacist role is evolving; however, several challenges remain in fully realising the potential of pharmacists. These include regulatory barriers, limited public awareness of pharmacists’ expanded roles, workforce capacity issues, and the need for ongoing professional development and training. Research studies are needed in the areas of generic prescribing, medicine adherence, intervention studies in community and hospital pharmacy practice, and on pharmacoeconomics and pharmacovigilance.

## 1. Background

### 1.1. Health and pharmacy indicators in Türkiye

As of 2024, the total Turkish population is about 85 million and life expectancy is around 78.6 years (TÜİK, 2024). The preventable mortality was recorded at 126 per 100,000 (OECD Health at a Glance 2023 Country Note: Türkiye, 2023). While the crude death rate which is the number of deaths per thousand persons was 6.7 per thousand in 2021, it was 5.9 per thousand in 2022. When the deaths were analyzed by causes, deaths from diseases of the circulatory system were the highest with 35.4% in 2022. This cause of death was followed by neoplasms with 15.2% and diseases of the respiratory system with 13.5% (TÜİK, 2022a).

As of 2022, Türkiye spent 4.0% of its Gross Domestic Product (GDP) on health (TÜİK, 2022b; WB, 2020). Türkiye spends $1827 per capita on health, less than the OECD average of $4986 (USD PPP). There are 2.2 practicing doctors per 1,000 population (OECD average 3.7); 2.8 practicing nurses (OECD average 9.2); and 0.41 practicing pharmacists (OECD average 0.82) in 2023 (Kıran & Akbolat, 2021; OECD Health at a Glance 2023 Country Note: Türkiye, 2023). As outlined in the Ministry of Health's 2023 Vision for Human Resources in Health, the demand for pharmacists is projected to reach 32,032 by the year 2023 in Türkiye (MoH, 2023). However, the current number of pharmacists has surpassed 41,000 (Arastaman et al., 2018).

The Turkish pharmaceutical market reached $3.5 billion in 2022 (IEIS, 2023). In Türkiye, community pharmacies are recognised as primary healthcare organisations (MoH, 2019). There are 28,465 community pharmacies in the country. In Europe, the ratio of community pharmacists per 10,000 people is 7.23, whereas in Turkey, this rate is 3.84 (Gül et al., 2023).

### 1.2. Pharmacy practice in Türkiye

In recent decades, the domain of pharmacy practice has broadened to include considerations of clinical, behavioural, economic, and humanistic aspects. Additionally, there is an emphasis on incorporating innovative practices such as health interventions and patient care services, often in collaboration with other healthcare professionals including physicians and nurses (Fernandez-Llimos et al., 2023).

Pharmacists hold a societal obligation to provide patient care, and identifying the most effective means of delivering this care involves adopting a scientific approach (Garcia-Cardenas et al., 2020). Although no universally accepted definition for pharmacy practice research exists, the International Pharmaceutical Federation Pharmacy Practice Special Interest Group (FIP PPR – SIG) defined it as ‘the scientific discipline that studies the different aspects of the practice of pharmacy and its impact on healthcare systems, medicine use, and patient care’ (FIP, 2024). Pharmacy practice and policy research, mainly tied to social and administrative pharmacy (Babar & Jamshed, 2008), form an integral part of health services research.

It entails the evaluation of pharmacy practice to enhance our understanding of medications, promote the shift towards evidence-based practices, and assist policymakers in creating and implementing new services (Koshman & Blais, 2011). It covers the areas associated with the distribution, selection, regulation, safety, efficacy, and quality of drugs, as well as pharmaco-epidemiology, access to medicines, pharmacoeconomics, pharmacovigilance (PV), and pharmaceutical care (PC) (Desselle, 2005). Research in pharmacy practice and policy is essential to improve the pharmacy profession and enhancing the broader healthcare system, particularly in low – and middle-income countries (LMICs).

The Turkish healthcare system has undergone significant transformation, particularly with the implementation of the Health Transformation Program (HTP) between 2003 and 2013. This aimed to expand universal health coverage, promote equity, and reduce disparities in healthcare services and health outcomes. This reform effort has led to substantial implications for primary healthcare in Türkiye, including community pharmacy practice (Akinci et al., 2012). As part of the programme, there were infrastructural improvements in health information technology, potentially influencing the adoption of electronic health records in Turkish hospitals, (Kose et al., 2020) which could have implications for healthcare system research and policy development in Türkiye.

### 1.3. Renewed interest in pharmacy practice and policy research in Türkiye

The scope of pharmacy practice and policy research in Türkiye is a topic of growing interest. The implementation of clinical pharmacy education in Türkiye has been observed and experienced, indicating progress in this area at the beginning of the 1990s (Bektay et al., 2020). Following the adoption of the law on pharmacy specialisation in 2014, clinical pharmacy specialisation training was started in 2018 as a postgraduate training programme. This training consists of theoretical courses, case presentations, intensive clinical environment training, and specialisation thesis stages for 3 years (Kara, Okuyan, et al., 2021). The first specialist clinical pharmacists started working in public hospitals as of May 2023.

Before the implementation of clinical pharmacy as a discipline in Türkiye, a group of social pharmacy scholars have been working in the field of pharmacy practice and policy since the establishment of the Department of Pharmacy Management in 1981 (The Department of Pharmacy Management at Ankara University, 2023). Most pharmacy practice research conducted by the Departments of Clinical Pharmacy in Türkiye is focused on pharmacist input in acute and chronic disease management, oncology pharmacy, hospital pharmacy, pharmacotherapy in general, pharmaceutical care, and rational drug use, especially in hospital settings (Kara, Kelleci Cakir, et al., 2021). Furthermore, the focal point of investigation within the domain of social pharmacy research predominantly revolves around the examination of the roles of pharmacists in pharmacy services, pharmacy management, and pharmacy education. A very few research studies have been undertaken to explore pricing, access, affordability of medicines, and pharmacoeconomy in Türkiye in the last five years (Atikeler et al., 2020; Guven, 2023; Mashaki Ceyhan et al., 2020; Oksuz et al., 2021; Tengiz et al., 2023; Topcuoglu & Arsava, 2021).

The researchers in Türkiye encounter barriers when conducting research in pharmacy practice and policy. These challenges include the absence of documentation especially in community settings, time constraints, regulatory restrictions or complex bureaucratic processes, insufficient financial support, inadequate motivation to participate into research among pharmacists and patients, limited collaboration with other healthcare professionals.

The government's role in supporting evidence-based policy development is crucial in addressing deficiencies in pharmacy practice. The recognition of the need for evidence-based public health policy is of vital importance (Ozcebe et al., 2018). This emphasis on evidence-based policymaking is essential for driving improvements in pharmacy practice and addressing healthcare deficiencies. However, there is a dearth of collaboration in supporting research initiatives aimed at addressing deficiencies in the healthcare system concerning pharmacy practice and policy in Türkiye. This shows the research gap, the need to identify research priorities and the collaborative linkages between researchers, practitioners, and policymakers (Malik et al., 2020).

Developing evidence-based policies is most effective when utilising local research data. In LMICs, however, policies often draw on data and success stories from more developed nations, overlooking the local context, realities of health service delivery, and economic differences (Ahmed et al., 2018). This fails to consider the specific local context, the intricacies of health service delivery, and the economic disparities within the country (Ahmed et al., 2018). A deficiency in evaluating data and information collected through health information systems to be used for evidence-based decision-making and health policy development and planning processes in Türkiye is needed (Okem & Cakar, 2015).

### 1.4. Rationale of the study

In the last 20 years, there is an interest in clinical pharmacy practice in Türkiye with emerging studies in this area. Though there are individual studies focusing on clinical pharmacy, medicines use, patient counselling, and compliance, social and behavioural aspects of pharmacy practice however, there is a lack of a comprehensive review portraying the overall situation. Despite the recent emergence of diverse pharmacy practice studies in Türkiye, a comprehensive assessment of their overall typology and impact has not been conducted thus far. Given this, there is a need for systematic reviews to harness the existing body of research and enable its influence on healthcare policymakers (Malik et al., 2020; Tacconelli, 2010). This will enhance their understanding of how findings impact professional practices and subsequent patient outcomes (Connor et al., 2023). A thorough assessment of the quality of available literature is also crucial to establishing a dependable knowledge foundation and ensuring that policies are formulated based on robust evidence (Gray & Suleman, 2015). By providing a comprehensive assessment of the current state of pharmacy practice in Türkiye, our study offers insights that are not only relevant to researchers and practitioners in Türkiye but also hold broader implications for LMICs facing similar healthcare challenges and barriers in pharmacy practice and policy research. By establishing a dependable knowledge foundation through a systematic review, this research can guide policymakers in formulating robust, evidence-based policies (Vogler et al., 2024). Moreover, the findings can inform future research directions and collaborative efforts, ultimately contributing to the advancement of pharmacy practice and healthcare outcomes in Türkiye. In this context, the present systematic review is planned.

This systematic review aims to (1) document and assess pharmaceutical policy and practice literature published within the last 5 years in Türkiye, (2) summarise the expected impact of published studies on policy and practice research in Türkiye.

## 2. Methods

The systematic review conducted according to the guidelines described in the Preferred Reporting Items for Systematic Reviews and Meta-Analyses: The PRISMA Statement.

### 2.1. Research strategy

A comprehensive search approach, incorporating Medical Subject Headings (MeSH) queries and free-text terms, was employed to locate pertinent literature related to pharmacy practice and policy in Türkiye. The search covered the period from January 1, 2019, to January 1, 2024, and involved electronic databases including PubMed, Medline Ovid, Scopus, ScienceDirect, Springer Link, PlosOne, and BMC. Internet search engines such as Google and Google Scholar were also employed in the search process. Additionally, internet search engines like Google and Google Scholar were utilised in the search process. The keywords used were: (pharmaceutical policy OR practice); AND (evidence OR impact OR rational OR rational use of medicines); AND (community pharmacy OR community pharmacies OR pricing OR access to medicines OR generic medicines OR medication OR healthcare OR care OR pharmacy); AND (Turkey OR Türkiye). The reference lists of pertinent articles were also reviewed to identify additional publications through backward citation.

### 2.2. Inclusion and exclusion criteria for selection of studies

The selection process focused on studies published in English and incorporating specific keywords. Two researchers (GG, AP) from the group independently conducted the initial screening of titles and abstracts. Relevant studies falling within predefined domains were included:

- Community pharmacy studies
- Pharmacovigilance
- Pharmaceutical policy
- Practice
- Medicine pricing
- Rational use
- Medicine access

Studies unrelated to the research scope and search criteria were excluded. The inclusion and exclusion criteria for the studies were detailed in Table 1.

**Table 1.** Inclusion and exclusion criteria.

**Inclusion criteria**
Studies published in electronic databases as scientific literature during 2019–2024
Full-text studies
All studies published in English
Studies showing quality evaluation criteria >75%
**Exclusion criteria**
Pilot studies, letters, commentaries and viewpoints, editorials, review articles
and animal clinical trials
Research not stating clear outcomes

### 2.3. Article selection and risk of bias (quality) assessment

**Figure 1.**
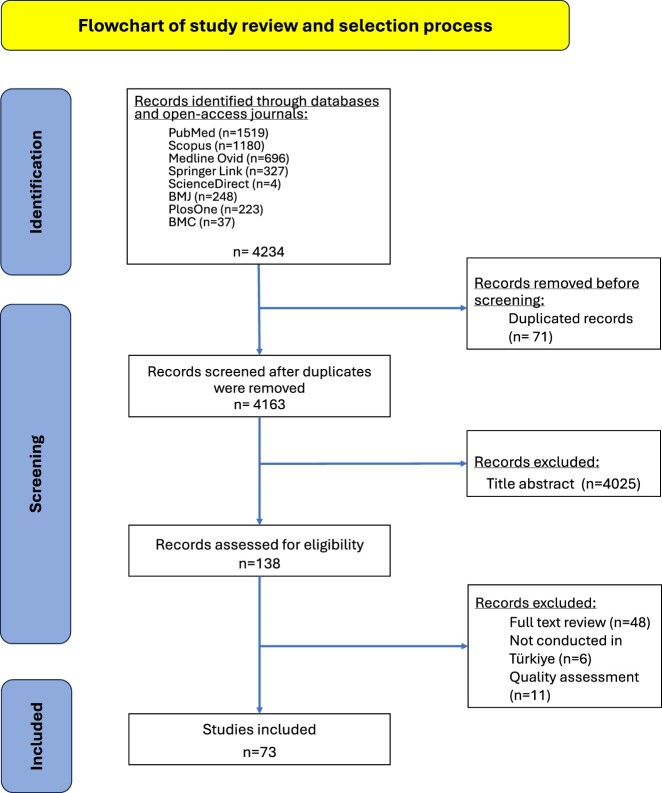
PRISMA flowchart of the study.

Initially, records were imported into Rayyan®, and duplicate entries were eliminated. The article selection process consisted of two steps. In the first step, the articles’ abstracts were examined in terms of inclusion and exclusion criteria after the duplicates were removed. In the second step, the appropriate articles were searched for full text and assessed by the two reviewers (GG, AP) in terms of inclusion and exclusion criteria. Inconsistencies highlighted by the reviewers were resolved in peer panel meetings amongst all authors. The flow diagram of the article selection process is provided in Figure 1.

The studies were evaluated and analysed for quality using Mixed Methods Appraisal Tool (MMAT) (Hong et al., 2018). The MMAT incorporates two primary screening criteria to evaluate the formulation of a distinct research question/objective and the suitability of the gathered data for addressing the research query. To be eligible for inclusion, studies must receive a positive assessment. Additionally, the tool features a set of specific questions designed to appraise the quality of studies within each type – qualitative, quantitative, and mixed methods.

Two independent reviewers (GG and AP) evaluated, and scored selected studies based on rating criteria. Following the established method for applying MMAT, a set of five questions tailored to each study design type is given a binary response of ‘yes’, ‘no’, and ‘can’t tell’. Studies are allocated 20 percentage points for each affirmative answer. The percentage assigned indicates the level of quality, with a score of 100% signifying that all quality criteria have been satisfied. Studies were chosen only when there was consensus on their quality between both reviewers (GG, AP). In cases of disagreement between the designated reviewers, resolution was achieved by involving a third independent reviewer (ZUDB).

### 2.4. Data extraction and analysis

The following information from the selected articles was then extracted and examined by the researchers, GG and AP, and ZB: (1) first author, (2) year of publication, (3) name of the journal, (4) study objective, (5) study design, (6) study setting, (7) sample size and (8) main findings of the study. A spreadsheet to record this information was completed for all selected articles and subsequently tabulated in Supplemental Appendix 1.

The groundwork for conducting thematic analysis on selected papers in pharmacy practice and policy research is rooted in existing literature (Babar & Jamshed, 2008; Desselle, 2005). Following the perspective outlined by Ryan and Bernard (Ryan et al., 2003), themes were identified to encapsulate the theoretical foundations, arguments, and conceptual relationships inherent in the problem statement, concepts, and analysis of each study. This process involved extracting themes based on a comprehensive understanding of the fundamental concepts presented in the studies. Both authors (GG, AP) independently categorised all papers, with multi-objective articles assigned to the category most closely aligned with their primary subject matter. The resulting classifications were compared, and cohesive classifications were consolidated into final themes during a group discussion. In instances of non-consensus or disagreement, a senior author (ZUDB) moderated the discussion and determined the resolution.

## 3. Results

As provided in the PRISMA diagram (Figure 1), the initial literature search from 2019 to 2024 resulted in 4166 potentially relevant citations. Of these, 71 were duplicates which were omitted. Of the 4234 remaining articles, 4163 were excluded based on irrelevant titles and/or abstracts. Subsequently, 138 studies were carefully examined to assess eligibility, and 65 articles were excluded at this stage for failing to present pertinent information. In the final grouping, 73 articles met the inclusion criteria and were selected for this review.

### 3.1. Characteristics of the selected studies

The main characteristics of the 73 studies are provided in Supplemental Appendix 1. Among the quantitative studies, nineteen studies were cross-sectional survey studies (Albayrak et al., 2021; Aydın et al., 2020; Bulut & Bilgener, 2022; Camcioglu et al., 2020; Çelebi et al., 2022; Ekmez & Ekmez, 2022; Ergun et al., 2019; Gulpinar & Uzun, 2022; Kara et al., 2020; Küçükali et al., 2021; Okuyan, Balta, et al., 2021; Okuyan, Bektay, et al., 2021; Oncu et al., 2021; Ozdemir et al., 2022; Ozturk et al., 2019; Tarhan & Arslan, 2023; Tengilimoğlu et al., 2020; Yazicioglu & Yardan, 2021; Yilmaz & Sencan, 2021), thirteen studies utilised prospective cross-sectional study designs (Acimis et al., 2022; Albayrak & Demirbas, 2023; Atac et al., 2022; Buyuker et al., 2023; Khan et al., 2023; Kirmizi Sonmez et al., 2023; Kockaya et al., 2020; Ozturk et al., 2021) (Akarsu OrunÇ & Arslan, 2023; Goker et al., 2022; Kuerec et al., 2023; Sumbul-Sekerci et al., 2022; Yilmaz & Bulut, 2023), eight were prospective interventional (Albayrak et al., 2022; Kandemir et al., 2021; Kara, Metan, et al., 2021; Okuyan, Ozcan, et al., 2021; Pehlivanli et al., 2023; Savas et al., 2021; Umar et al., 2020; Yildirim et al., 2023), three were prospective observational (DemİRcİOglu Akyilmaz et al., 2022; Enver et al., 2023; Yılmaz et al., 2020), three were none randomised controlled (Apikoglu et al., 2022; Dumlu et al., 2022; Uzun et al., 2019), one was randomised controlled (Bektay et al., 2022), four studies were retrospective (Alp et al., 2021; Ayhan & Sancar, 2023; Bulut et al., 2019; Idil et al., 2021), three were retrospective cross-sectional studies (Aksoy et al., 2021; Ozdamar & Ozdamar, 2021; Pehlivanlı et al., 2022), one was a retrospective cohort study (Güzeloğlu & Karacı, 2022), one used both retrospective and prospective cross-sectional study techniques (Şahin et al., 2022), five was a descriptive study (Atikeler et al., 2020; Bayram et al., 2021; Ersoy & Engin, 2019; Ertuna et al., 2019; Kockaya et al., 2021). Two was a methodological study (Cengiz & Ozkan, 2022; Hagens et al., 2021). A qualitative study design was adopted in five studies (Aci et al., 2022; Erdoğan et al., 2021; Fedai Kayin et al., 2023; Gülpınar et al., 2021; Westerling et al., 2020). The remaining one study applied a mixed-method design (Mashaki Ceyhan et al., 2020).

Most studies, that is, twenty-two, were published in 2021. Out of the 47 remaining studies, seventeen were published in 2022, fifteen each in 2023, nine in 2020, and six in 2019. Five studies (Apikoglu et al., 2022; Dumlu et al., 2022; Okuyan, Balta, et al., 2021; Savas et al., 2021; Umar et al., 2020) were published in the *International Journal of Clinical Pharmacy*, four studies each were published in the *Turkish Journal of Pharmaceutical Science* (Enver et al., 2023*;* Kara et al., 2020*;* Pehlivanlı et al., 2022*;* Yilmaz & Sencan, 2021) and *Journal of Research in Pharmacy* (DemİRcİOglu Akyilmaz et al., 2022*;* Kuerec et al., 2023*;* Şahin et al., 2022*;* Yilmaz & Bulut, 2023), three studies were published in *Vaccines* (Buyuker et al., 2023*;* Gulpinar & Uzun, 2022*;* Hagens et al., 2021), and two studies each were published in *Journal of the American Pharmacists Association* (Gülpınar et al., 2021*;* Okuyan, Ozcan, et al., 2021), *Brazilian Journal of Pharmaceutical Science* (Bulut & Bilgener, 2022*;* Goker et al., 2022), and *Ankara Üniversitesi Eczacılık Fakültesi Dergisi* (Akarsu OrunÇ & Arslan, 2023; Ayhan & Sancar, 2023).

### 3.2. Research domains identified

Through the rigorous thematic content analysis previously outlined, seven research domains were developed from the selected literature: drug utilisation and rational drug use, the emerging role of pharmacist, access to medicines and generic medicines, community pharmacy practice, pharmacovigilance/adverse drug reactions, and pharmacoeconomic studies. The key focus of the studies on pharmacy practice and policy published in the last five years in Türkiye across research domains is summarised in Table 2

**Table 2.** The key focus of the studies on pharmacy practice and policy published in the last five years in Türkiye across research domains.

Theme	Main domain
Appropriate use of antibiotics, access to antibiotics, communication with and trust in physicians for appropriate use of antibiotics (1) Ordering an antibiogram test before prescribing (3) Physicians pay attention to prescribing effective, safe, appropriate drugs (3) Attitudes of healthcare workers towards COVID-19 vaccination (4) COVID-19 vaccine hesitancy among pregnant women is related to factors of being nulliparous, having a chronic disease, having a university degree, working as a professional healthcare provider, and having a history of death due to COVID-19 in relatives and acquaintances significantly (7) Painkiller selection and the level of knowledge are affected by demographic features and level of knowledge about them (10) Antibiotic use without prescription can be seen in the country. Publication of treatment guidelines can be the best way to regulate antibiotic use (11) Rational antibiotic use, high antimicrobial resistance rates, and publishing local cumulative antibiograms are very important for AMS, each hospital should establish its stewardship programme and revise it. Improvement in rational antibiotic use is hard to achieve without multidisciplinary involvement (12) Governmental interventions at the national level led to a reduction in antibiotic prescriptions at the primary healthcare level (14) The prescribing performances of PCPs are not rational enough in terms of drug selection and prescription content (16) Nonpharmacological approaches against polypharmacy, such as deprescribing in geriatric practice are common. CGA and Beers criteria help healthcare professionals to detect PIMs (18) Education on COPD management increased patients’ self-care agency (19) Community pharmacists should be trained to develop skills in identifying and reducing PIMs in older patients (20) Children's satisfaction and confidence should be improved as patients in adherence to drug therapy (21) High rate of PIM use and polypharmacy in elderly hospitalized patients (26) Polypharmacy is an important risk for anticholinergic burden (30) Pharmacists play important role in patients’ compliance. Lack of methods to increase patient compliance in pharmacy education (35) The drug usage habits of the elderly were not found to be rational (40) Patient education has a positive effect both on patient adherence and hypertension illness perception (47) Attitudes of hospital pharmacists towards protective behaviour from COVID-19 (49) Consumer awareness of the rational use of medicines is at a low level. Lack of awareness of waste management, a decrease in the rate of self-medication as age increases (50) Lack of knowledge about antibiotic use among parents (65) Tool development aiming to rational drug use (66)	**Drug Utilisation, Rational Drug Use (22 articles)**
When the knowledge level of the physicians increased, negative opinions and prescribing attitudes regarding the effectiveness, quality, and safety of generic medicines decreased (5) No generic prescribing in Turkey (9) Pharmaceutical pricing and reimbursement policies have been taken place to control pharmacy/medicine expenditure (17) The pharmaceutical ITS (Turkish Pharmaceutical Track & Trace System) application has provided advantages such as facilitating procedures for pharmacies, as well as providing safe production, distribution, and access of medicines to the patients (34) To exhibit the nationwide trend of generic medicine use in primary care (41) Pharmaceutical Track and Trace System is found useful especially for providing drug safety and facilitating and accelerating the recall of drugs (44) Health insurance is increasing prescription medicine use (51) Family physicians are satisfied with the application of e-prescriptions and identified positive effects on their work and processes (52) Accessibility to family health physicians increases polypharmacy risk. People who have no health insurance experience less polypharmacy due to the risk of inadequate treatment (53) Priorities of patients are faster access to new medicines with low cost and engagement of regulatory decision-making processess (56) The policy called ‘Medicines Broad from Abroad’ for accessing orphan drugs is insufficient due to pricing and reimbursement conditions in Turkey (57) Lack of policy regarding access to orphan drugs (58) Policy makers should perform a systemic analysis of market, political, economic, technical, and legal factors before implementing any pharmaceutical track and trace system (73) To achieve the intended outcomes, system design must align with the goals of implementation (e.g.to tackle fraud, reduce falsified medicine, minimise shortages). The system must be feasible in the local political and economic context (73)	**Access to medicines and generic medicines policy (13 articles)**
Community pharmacist-led pharmaceutical care services significantly improved patient-related outcomes (such as medication adherence, beliefs about medication, and QoL) in older adults with noncommunicable diseases (2) Intention to provide vaccination services has been affected by patient-related attitude toward PDV services, attitudes toward negative consequences of PDV services, and subjective norms about PDV services (13) Perceived enablers and barriers related to pharmaceutical services and burnout level during the COVID-19 pandemic (15) CPs have a positive attitude toward counselling in celiac disease, but there are some deficiencies in knowledge and practice (25) Although community pharmacists can help improve the health outcomes of patients with asthma, COPD, diabetes, and hypertension, they are facing barriers in collecting patient data and documenting (31) Misleading information. More than half of the pharmacists trust social media and government resources regarding COVID-19 (39) Providing pharmacy services based on conscious and religious beliefs (42) Community pharmacists could cope with the psychological effects of the pandemic (45) Knowledge and attitude of pharmacists and pharmacy technicians on emergency contraception pills (ECP) (55) Community pharmacists are facing barriers during pharmaceutical care provision (59) A measurement tool to explore pharmacist counselling for dementia (67) Pharmacists’ knowledge and attitude about vaccines (69)	**Community Pharmacy Practice (12 articles)**
Assessment of adherence to cancer-associated venous thromboembolism guideline and pharmacist's impact on anticoagulant therapy (6) Medication reconciliation service solved unintentional medication reconciliation discrepancies during COVID-19 (24) Clinical pharmacists’ interventions for identifying, preventing, and resolving DRPs (27, 54, 61) Drug related problems (DRPs) decrease with the interventions of clinical pharmacists in hospital settings (28, 38, 61, 62, 64) Pharmacists have an impact to make important contributions on increasing patients’ awareness and knowledge regarding Diabetes Mellitus (29) To identify the prevalence of potentially inappropriate medication use (PIMU) in adults above the age of 65 with chronic kidney disease (CKD) (32) Positive impact of the clinical pharmacist-led hypertension screening programme (36) Patient satisfaction achieved by provision of drug information regarding neuropathic pain by a clinical pharmacist (37) The integration of a clinical pharmacist into the pain management team may have a positive impact on the patients’ knowledge, concerns, and misconceptions about opioids, which may improve adherence and effective pain management in cancer treatment (60) Clinical pharmacist-led stewardship programme for the appropriate use of acid suppression therapy improved the rate of appropriate proton pump inhibitor use and reduced the potentially inappropriate proton pump inhibitor use during the hospital stay (63) A clinical pharmacist’s evaluation of fungal infections and assessment of may help to increase the quality of antifungal stewardship (AFS) (68)	**Emerging Role of Pharmacist (16 articles)**
Barriers seen in adverse drug reaction reporting system (TUFAM) and pharmacovigilance contact points among healthcare providers (22, 46, 48) To assess short-term adverse events following immunization (23) Side effects of antibiotics are common (33) Healthcare providers prefer to report adverse drug reactions to another physician rather than TUFAM (47)	**Pharmacovigilance /Adverse Drug Reactions (6 articles)**
A higher tendency of prescribing for no clear indication are also more likely to produce costly prescriptions (8) Cost effectiveness of COVID-19 vaccination (43) Cost-effectiveness of Omalizumab compared with standard-care of therapy in patients with severe asthma (70) Medicines used for tricuspid regurgitation has great importance in widespread use based on cost-effectiveness analysis (71) Remdesivir used in COVID-19 is associated with higher QALYs and lower costs (72)	**Pharmacoeconomic study (5 articles)**

*AMS* Antimicrobial Stewardship, *AFS* Antifungal Stewardship, *CGA* Comprehensive Geriatric Assessment, *CKD* Chronic Kidney Disease, *COVID-19* Coronavirus Disease-19, *COPD* Chronic Obstructive Pulmonary Disease, *CP* Community Pharmacist, *CPR* Clinical Pharmacy Resident, *DRP* Drug-Related Problem, *ECP* Emergency Contraception Pills, *ITS* Turkish Pharmaceutical Track & Trace System, *PCP* Primary Care Physician, *PIM* Potentially Inappropriate Medication, *PIMU* Potentially Inappropriate Medication Use, *TUFAM* Türliye Farmakovijilans Merkezi, *QALY* Quality-Adjusted Life-Year

#### 3.2.1. Domain 1 – drug utilisation and rational drug use

Most of the studies included provided insights into different facets of medication utilisation. The most investigated topic was medication usage behaviour of public (Albayrak et al., 2021; Camcioglu et al., 2020; Cengiz & Ozkan, 2020; Ekmez & Ekmez, 2022; Ozturk et al., 2021; Tengilimoğlu et al., 2020; Westerling et al., 2020; Yazicioglu & Yardan, 2021), followed by prescribing practice of physicians (Acimis et al., 2022; Aksoy et al., 2021; Atac et al., 2022), assessment of rational drug use (Albayrak & Demirbas, 2023; Kuerec et al., 2023; Sumbul-Sekerci et al., 2022), and knowledge, attitude, and practice of pharmacists and other healthcare professionals towards COVID-19 vaccination (Aci et al., 2022) and evaluation of protective behaviours from COVID-19 (Kara et al., 2020). A few studies classified as intervention to improve medication use (Alp et al., 2021; Idil et al., 2021; Yildirim & Kasikci, 2023). Nearly 4% of the included studies focused on patient adherence. Two articles were about assessing the role of pharmacists in patient adherence (Çelebi et al., 2022; Fedai Kayin et al., 2023), followed by only one study related to an intervention towards increasing patient knowledge in hypertension to develop adherence (Yılmaz et al., 2020).

#### 3.2.2. Domain 2 – the emerging role of pharmacist

Around 21% of the included studies were about the role and impact of clinical pharmacists in various healthcare settings. In this domain, studies covered the diverse roles of clinical pharmacists in healthcare reported their contribution to adherence to guidelines (Kandemir et al., 2021), patient satisfaction (Goker et al., 2022), interventions leading to reduction of drug related problems (Albayrak et al., 2022; Ayhan & Sancar, 2023; Ertuna et al., 2019; Pehlivanli et al., 2023; Şahin et al., 2022; Umar et al., 2020), medication reconciliation service (Enver et al., 2023), pharmacists’ role in identifying potentially inappropriate medication use (Pehlivanlı et al., 2022), improvement of treatment outcomes through patient education (Savas et al., 2021; Yilmaz & Bulut, 2023), and stewardship programmes (Dumlu et al., 2022; Kara, Metan, et al., 2021). The research articles concerning the emerging role of pharmacist were covering different pharmacy settings. Hospital clinic was the dominant setting in which pharmacy practice research studies were conducted (Albayrak et al., 2022; Bektay et al., 2022; Dumlu et al., 2022; Enver et al., 2023; Ertuna et al., 2019; Goker et al., 2022; Kandemir et al., 2021; Kara, Metan, et al., 2021; Pehlivanli et al., 2023; Pehlivanlı et al., 2022; Şahin et al., 2022; Umar et al., 2020), this was followed by community pharmacy (DemİRcİOglu Akyilmaz et al., 2022; Yilmaz & Bulut, 2023).

#### 3.2.3. Domain 3 – access to medicines and generic medicines policies

Accessing to medicines and generic medicines were investigated in 13 studies. These studies encompassed pharmaceutical pricing and reimbursement policies (Kockaya et al., 2020; Mashaki Ceyhan et al., 2020), advantages of the Turkish Pharmaceutical Track and Trace System (ITS) (Bulut & Bilgener, 2022; Erdoğan et al., 2021; Parmaksız et al., 2020), access to orphan drugs (Atikeler et al., 2020; Kockaya et al., 2021), preferences of physicians in prescribing generic medicines (Bayram et al., 2021; Oncu et al., 2021), practicing generic prescribing (Ozdamar & Ozdamar, 2021), the impact of health insurance in accessing to medicines (Ersoy & Engin, 2019; Ozturk et al., 2019), and application of electronic prescriptions (Bulut et al., 2019).

#### 3.2.4. Domain 4 – community pharmacy practice

Seven studies out of 12 articles were related to CPs’ knowledge, attitude, and practice toward pharmacy services (Akarsu OrunÇ & Arslan, 2023; Gulpinar & Uzun, 2022; Gülpınar et al., 2021; Okuyan, Bektay, et al., 2021; Ozdemir et al., 2022; Tarhan & Arslan, 2023; Uzun et al., 2019; Yilmaz & Sencan, 2021). Two studies were about evaluating interventions in community pharmacies (Apikoglu et al., 2022; Okuyan, Ozcan, et al., 2021). A study assessed current pharmacy practices (Okuyan, Balta, et al., 2021), followed by an evaluation of the pandemic preparedness of community pharmacists (Küçükali et al., 2021).

#### 3.2.5. Domain 5 – pharmacovigilance/adverse drug reactions

In total 6 studies investigated pharmacovigilance and adverse drug reactions. Barriers to reporting adverse drug reactions were covered in four studies (Aydın et al., 2020; Ergun et al., 2019; Khan et al., 2023; Yılmaz et al., 2020), followed by monitoring and surveillance studies (Buyuker et al., 2023; Güzeloğlu & Karacı, 2022).

#### 3.2.6. Domain 6 – pharmacoeconomic studies

Almost 6% of the included studies were about the pharmacoeconomic evaluation of various medicines and vaccinations (Hagens et al., 2021; Oksuz et al., 2021; Tugay et al., 2023).

## 4. Discussion

This study aimed to explore the recent literature on pharmacy practice and policy research in Türkiye spanning the last five years. Through a thematic synthesis of the literature, seven key domains were identified, each representing a distinct research focus. The subsequent sections delve into each of these domains, providing comprehensive coverage of the identified research areas.

Regarding advanced methodologies, the literature reviewed underscores the utilisation of mixed methods, with qualitative and cross-sectional simulated study designs increasingly prevalent. Despite the enhancement in the standard of pharmacy practice and policy research in Türkiye, there remains a paucity of literature examining the influence of pharmacy research, policy, and pharmacoeconomics, which represent emerging fields in pharmacy practice. This gap is possibly attributed to limited access to diverse patient data, inadequate establishment of relationships and collaborations with other healthcare professionals, particularly medical doctors, and the limited number of researchers dealing with these fields.

### 4.1. Drug utilisation and rational drug use

Although examining the socio-behavioural aspects of patients in drug use is the common focus of different authors, there are almost no studies on the self-management of diseases. Consistent with this result, it was found that the number of studies on adherence was very limited (Fedai Kayin et al., 2023). A limited number of studies discussed the interventions and solutions to improve medication use (Yildirim & Kasikci, 2023; Yılmaz et al., 2020). This may be because community pharmacists in Türkiye are more responsible for medicine distribution and dispensing. Similarly, majority of hospital pharmacists are responsible for procuring and dispensing medicines. Another reason may be that emerging clinical pharmacy practices occur in the hospital environment and studies generally focus on inpatients and outpatient patients are underestimated.

Approximately 30% of the studies on rational drug use focus on the use of antibiotics (Acimis et al., 2022; Aksoy et al., 2021; Albayrak et al., 2021; Alp et al., 2021; Camcioglu et al., 2020; Westerling et al., 2020). The Ministry of Health of Türkiye has established two antimicrobial stewardship programmes (ASPs); the first targets hospitals and the second the community. The first was the National Hospital Antimicrobial Restriction Programme (NARP), which was legislated by the Ministry of Health in 2003. The objective of this mandatory programme was to reduce hospital antimicrobial use by mandating preauthorization from an infectious disease specialist before using several broad-spectrum antibiotics (e.g. carbapenems, glycopeptides, piperacillin/tazobactam, ceftazidime, and cefepime) (Isler et al., 2019). An audit and feedback system targeted all FP prescriptions to reduce antibiotic prescriptions, which accounted for 35% of FP prescriptions in 2011. Seminars and workshops were used to train healthcare workers. As part of the same programme, the Rapid Strep Test was introduced to primary care as an application of point-of-care tests. Public awareness of prudent antibiotic use was a secondary goal of the programme (Isler et al., 2019). Until 2013, antibiotics could be purchased from pharmacies without a prescription in Türkiye. In 2013, the Ministry of Health restricted the purchase of antibiotics from community pharmacies without a prescription (Emre-Aydıngöz & Lux, 2021). Total systemic antibacterial drug consume in Türkiye, which was 41.1 DID in 2013, decreased to 35.5 and 31.0 DID in 2017 and 2018, respectively. While Türkiye had the highest antibiotic consumption among OECD countries in 2013, this consumption decreased by 24.6% in 2018 (Emre-Aydıngöz & Lux, 2021). However, despite all these interventions, it has been stated that antibiotic resistance rates are high in Türkiye (OECD, 2018). This may be due to the fact that not all AMS components can be implemented at national level. There is a need for a coordinated and feedback-driven national policy in which all AMS components are implemented, and for studies to evaluate the effectiveness of these policies.

### 4.2. The emerging role of pharmacists

Pharmacists are increasingly taking on new and expanded roles within the healthcare system. Studies have shown that pharmacists play a crucial part in various aspects of healthcare delivery. For instance, research has highlighted the importance of pharmacists in promoting rational antimicrobial use (Kara, Metan, et al., 2021). This is particularly relevant in a country like Türkiye, where antibiotic resistance is a growing concern. Moreover, the collaboration between pharmacists and other healthcare professionals has been recognised as essential for ensuring medication safety and effectiveness (Albayrak et al., 2022; Bektay et al., 2020; Pehlivanli et al., 2023; Şahin et al., 2022; Umar et al., 2020). Pharmacists have demonstrated improved outcomes in different conditions (Albayrak et al., 2022; Ayhan & Sancar, 2023; DemİRcİOglu Akyilmaz et al., 2022; Dumlu et al., 2022; Goker et al., 2022; Kara, Metan, et al., 2021; Pehlivanli et al., 2023; Şahin et al., 2022; Umar et al., 2020).

Overall, the emerging roles of pharmacists in Türkiye reflect a broader trend toward recognising their expertise beyond traditional dispensing functions. As pharmacists continue to expand their scope of practice and involvement in various healthcare settings, their impact on patient care and public health outcomes is becoming increasingly evident. Through their expanded responsibilities in medication therapy management, patient counselling, and interdisciplinary collaboration, pharmacists are poised to make valuable contributions to the Turkish healthcare system, driving improvements in patient outcomes, medication safety, and healthcare efficiency.

Despite the positive implications, several challenges remain in fully realising the potential of pharmacists in Türkiye. These include regulatory barriers (Gulpinar & Uzun, 2022), limited public awareness of pharmacists’ expanded roles, workforce capacity issues (TC. Sağlık Bakanlığı. Sağlıkta İnsan Kaynakları 2023 Vizyonu, 2011), and the need for ongoing professional development and training (Deniz-Ulutaş et al., 2024). Addressing these challenges requires concerted efforts from policymakers, healthcare institutions, professional organisations, and educational institutions to create enabling environments, develop supportive policies and guidelines, and invest in workforce development and training programmes. Moreover, there are several points that can be considered in order to develop pharmacy practice that might have an impact on pharmacists’ involvement in healthcare in Türkiye (Babar, 2021).

### 4.3. Access to medicines and generic medicines policies

Access to medicines and generic medicines were included in many research studies, the most frequent topic was the pricing and reimbursement policies. This was followed by the studies evaluating the Turkish Pharmaceutical Track & Trace System (ITS) in Türkiye. The implementation of ITS has yielded several advantages, including streamlined pharmacy procedures and enhanced safety and accessibility of medicines for patients (Bulut & Bilgener, 2022). These findings underscore the importance of investing in technological solutions to improve medication traceability, authenticity, and distribution processes (Kumar, 2023). However, there was a shortage of studies examining the sustained impact of ITS on medication safety, distribution efficiency, and accessibility for patients, including evaluating their effectiveness in preventing medication shortages and counterfeit medicines (Babar, 2024).

Only one study mentioned that there are no generic prescribing practices in Türkiye. Generic prescribing plays a crucial role in improving access to medicines by increasing affordability, promoting cost-effectiveness, and enhancing medication accessibility for patients (Roy & Rana, 2018). With ever increasing costs in healthcare, quality use of generic medicines can significantly reduce expenditures on medicines (Babar et al., 2014).

The studies are needed on exploring the attitudes, beliefs, and perceptions of various stakeholders towards generic medicines, including factors influencing prescribing decisions, and concerns about medication quality, and preferences for branded versus generic medications. The research is also needed on evaluating cost-effectiveness and economic benefits of generic prescribing policies, including assessments of healthcare expenditure, cost savings, and return on investment for healthcare payers and providers.

### 4.4. Community pharmacy practice

The fourth most utilised topic was community pharmacy practices. Studies investigating developing and implementing pharmacy services are needed in community pharmacies. A similar result was found in a study demonstrating the results from several Middle East countries (Obaid et al., 2022). Most of the studies explored the knowledge, attitudes, and practices of CPs toward current and possible advanced services and challenges in service provision. The least used study design in community pharmacy practice research was interventional research to evaluate the effectiveness of pharmacist-led interventions, patient counselling programmes, or other pharmaceutical care services. While pharmacy practice is established worldwide, variations exist in practice among different countries (Babar & Jamshed, 2008). However, there is limited literature on the standard of pharmacy practice in LMICs including Türkiye, where such services have yet to be evaluated (Appiah-Num Safo et al., 2022). Most pharmacists in Türkiye work in community-based settings, with a significant portion of their time dedicated to medication dispensing activities. They are not able to spend their most of time with patients, providing patient counselling services due to various reasons (Gulpinar & Uzun, 2022), which still needs to be improved to be aligned with international standards (Joint FIP/WHO guidelines on good pharmacy practice: Standards for quality of pharmacy practice, 2011). According to a study conducted in 2019, the knowledge of community pharmacists regarding emergency contraception pills to provide sufficient counselling needed to be improved, and a lack of skills in taking patient medication history was identified (Uzun et al., 2019). The renewed interest in pharmacy practice and policy research in Türkiye and a lack of a sufficient number of clinical and social pharmacy researchers to collaboratively perform intervention studies in community pharmacies might be an issue to be considered. Moreover, there is a lack of studies exploring patient perspectives, preferences, and experiences with community pharmacy services, including factors influencing their utilisation of services, barriers to access, and desired improvements in care delivery.

### 4.5. Pharmacovigilance/adverse drug reaction

Within the scope of pharmacovigilance activities, the Turkish Drug Adverse Effects Monitoring and Evaluation Center was established for the first time in Türkiye in 1985. Later, parallel to the regulation published in 2005 the name of this centre was changed to Turkish Pharmacovigilance Center (TUFAM) (Ergün, 2019). TUFAM is a national pharmacovigilance centre operating under the Ministry of Health. There has been limited research on pharmacovigilance. When the results of the studies were evaluated, the knowledge of healthcare professionals on pharmacovigilance in Türkiye, adverse drug reactions reporting, and TUFAM should be developed and backed up with research (Aydın et al., 2020; Ergun et al., 2019; Khan et al., 2023; Yılmaz et al., 2020). This lack of knowledge may be due to a lack of time and interest by healthcare professionals and a lack of training and courses on adverse drug reaction reporting and pharmacovigilance. There is a need for more studies to increase the awareness of healthcare professionals on this issue.

Unfortunately, there are no studies investigating pharmacists’ experiences on this subject. Since they do not have a reporting habit, no work can be done on this issue. Moreover, studies evaluating ADR reporting behaviours among pharmacists and other healthcare professionals highlight the need for targeted educational interventions and system-level improvements to enhance pharmacovigilance practices and ensure the timely detection and reporting of medication-related risks (Andrade et al., 2020; He et al., 2018; Phougat et al., 2024). Furthermore, pharmacists’ involvement in pharmacovigilance research fosters collaboration with regulatory agencies, pharmaceutical industry stakeholders, and healthcare organisations, facilitating the development of robust pharmacovigilance systems and the implementation of evidence-based interventions to promote patient safety and optimise medication use (Phougat et al., 2024) (Hussain et al., 2022).

### 4.6. Pharmacoeconomic studies

Studies in this domain focused on the economic impact of various medications and the assessment of the cost-effectiveness of different medications. There seemed to be a lack of specific pharmacoeconomic studies evaluating the cost-effectiveness, cost-minimization, or cost–benefit of pharmacist-led programmes or interventions not just for chronic disease management but also for self-care management. Consistent with this result, it was found that the number of studies on pharmacoeconomics was very limited (Obaid et al., 2022). Several studies might be conducted for specific conditions like diabetes, depression, and antimicrobial stewardship to highlight the importance of pharmacist involvement in improving patient outcomes, medication adherence, and overall quality of care. The cost savings associated with improved disease control, reduced hospitalisations, and decreased medication-related problems can help policymakers make informed decisions about allocating resources to support pharmacy services and initiatives (Schultz et al., 2021; Tonin et al., 2021).

The lack of pharmacoeconomic studies could be attributed to several factors. One possible reason might be the limited prioritisation of pharmacoeconomic research within the healthcare system in Türkiye (Alzarea et al., 2022). The focus may be more on immediate clinical outcomes and service delivery rather than conducting in-depth economic evaluations of pharmacy services. Furthermore, the absence of standardised pharmacoeconomic guidelines for conducting pharmacoeconomic evaluations in the Turkish healthcare setting is seen as a barrier (Yumrukaya et al., 2022). Moreover, the expertise and training in pharmacoeconomics among pharmacy professionals and researchers in Turkey is limited, leading to a gap in conducting these economic evaluations (Alzarea et al., 2022).

### 4.7. Implications for policy and practice

This work has potential implications for policy and practice as it has identified key themes and areas where the further work needed to be done. This is in all areas of policy and practice including access to medicines, use of medicines and medicines management. This work has highlighted some of the potential challenges as well as the need for a pharmacy practice research strategy for Türkiye. This has been done in several other countries including Malaysia (MoH, 2024). This mapping exercise is very useful to build further work in the areas of medicines selection, improving the quality use of medicines, as well as pharmaceutical care. This will be helpful in improving patient health outcomes in Türkiye. This work has generated several priotry research questions for pharmacy and medicines policy in Türkiye. To understand the impact of pharmacy services in Türkiye, there is an urgent need to develop additional pharmacy services and to evaluate not just their intervention outcomes but also their implementation outcomes to provide sustainability and uptake of these services. The second urgent topic may be evaluating cost-effectiveness and economic benefits of various interventions and policies such as generic prescribing policies, pharmacy services.

### 4.8. Strengths and limitations

With the fast-changing world where the use of medicines is on the rise, this is the first systematic review documenting the pharmaceutical situation in the Türkish context. The review elaborates on a growing number of studies in the areas of and has identified gaps where the number of studies including pharmacoeconomics and pharmacovigilance.

The limitation of this work is that it only looked at published literature. The grey literature and unpublished reports were not screened.

## 5. Conclusion

The pharmacist role is evolving; however, several challenges remain in fully realising the potential of pharmacists. These include regulatory barriers, limited public awareness of pharmacists’ expanded roles, workforce capacity issues, and the need for ongoing professional development and training. Though there is some evidence of growing pharmacy practice and policy research studies in Türkiye. There are several areas where the gaps are identified, and more work is needed in those areas. Research studies are needed in the areas of generic prescribing, medicine adherence, intervention studies in community and hospital pharmacy practice, and on pharmacoeconomics and pharmacovigilance.

## Supplementary Material

Appendix 1.xlsx

Figure.pptx

## References

[CIT0001] Aci, O. S., Kackin, O., Karaaslan, S., & Ciydem, E. (2022). Qualitative examination of the attitudes of healthcare workers in Turkey regarding COVID-19 vaccines. *International Journal of Nursing Knowledge*, *33*(2), 136–146. 10.1111/2047-3095.12342 34357685 PMC8441789

[CIT0002] Acimis, N. M., Yilmaz, I., Tekindal, M. A., & Kilic, R. (2022). Rational use of drugs in healthcare services: A sample of tertiary hospital. *European Review for Medical & Pharmacological Sciences*, *26*(5), 1701–1707. 35302219 10.26355/eurrev_202203_28239

[CIT0003] Ahmed, A., Tanveer, M., Siddiqui, A., & Khan, G. M. (2018). Bridging the gap for clinical pharmacist in developing countries like Pakistan. *Journal of the College of Physicians and Surgeons Pakistan*, *28*(3), 229–232. 10.29271/jcpsp.2018.03.229 29544583

[CIT0004] Akarsu OrunÇ, E., & Arslan, M. (2023). Evaluation of knowledge, attitudes, and practices of community pharmacists toward celiac disease. *Ankara Universitesi Eczacilik Fakultesi Dergisi*, *47*(3), 23–23. 10.33483/jfpau.1330731

[CIT0005] Akinci, F., Mollahaliloglu, S., Gursoz, H., & Ogucu, F. (2012). Assessment of the Turkish health care system reforms: A stakeholder analysis. *Health Policy*, *107*(1), 21–30. 10.1016/j.healthpol.2012.05.002 22652336

[CIT0006] Aksoy, M., Isli, F., Kadi, E., Varimli, D., Gursoz, H., Tolunay, T., … Alp Mese, E. (2021). Evaluation of more than one billion outpatient prescriptions and eight-year trend showing a remarkable reduction in antibiotic prescription in Turkey: A success model of governmental interventions at national level. *Pharmacoepidemiology and Drug Safety*, *30*(9), 1242–1249. 10.1002/pds.5311 34155708

[CIT0007] Albayrak, A., Basgut, B., Bikmaz, G. A., & Karahalil, B. (2022). Clinical pharmacist assessment of drug-related problems among intensive care unit patients in a Turkish university hospital. *BMC Health Services Research*, *22*(1), 79. 10.1186/s12913-022-07494-5 35033079 PMC8761343

[CIT0008] Albayrak, A., & Demirbas, H. (2023). Evaluation of potentially inappropriate medications use and medication complexity in elderly patients applying to community pharmacy in Turkey. *BMC Geriatrics*, *23*(1), 655. 10.1186/s12877-023-04381-4 37833671 PMC10571236

[CIT0009] Albayrak, A., Karakas, N. M., & Karahalil, B. (2021). Evaluation of parental knowledge, attitudes and practices regarding antibiotic use in acute upper respiratory tract infections in children under 18 years of age: A cross-sectional study in Turkey. *BMC Pediatrics*, *21*(1), 554. 10.1186/s12887-021-03020-4 34872522 PMC8647354

[CIT0010] Alp, E. E., Oncul, A., Dalgic, N., Akgun, C., Aktas, E., & Bayraktar, B. (2021). Antibiotic stewardship program experience in a training and research hospital. *SiSli Etfal Hastanesi Tip Bulteni / The Medical Bulletin of Sisli Hospital*, *55*(2), 253–261. 10.14744/SEMB.2020.96337 PMC8298082 34349604

[CIT0011] Alzarea, A. I., Khan, Y. H., Alanazi, A. S., Butt, M. H., Almalki, Z. S., AlAhmari, A. K., … Mallhi, T. H. (2022). Barriers and facilitators of pharmacoeconomic studies: A review of evidence from the Middle Eastern countries. *International Journal of Environmental Research and Public Health*, *19*(13). 10.3390/ijerph19137862 PMC9265831 35805521

[CIT0012] Andrade, P. H. S., de Almeida, A. C. B., Dos Santos, A. K. S., Lobo, I. M. F., da Silva, F. A., & da Silva, W. B. (2020). Challenges to the consolidation of pharmacovigilance practices in Brazil: Limitations of the hospital pharmacist. *Therapeutic Advances in Drug Safety*, *11*, 2042098620933748. 10.1177/2042098620933748 32864089 PMC7430076

[CIT0013] Apikoglu, S., Selcuk, A., Ozcan, V., Balta, E., Turker, M., Albayrak, O. D., … Uney, A. (2022). The first nationwide implementation of pharmaceutical care practices through a continuing professional development approach for community pharmacists. *International Journal of Clinical Pharmacy*, *44*(6), 1223–1231. 10.1007/s11096-022-01413-8 35699862 PMC9194772

[CIT0014] Appiah-Num Safo, A. A., Okoro, O. N., & Attakorah, J. (2022). Perceptions of healthcare providers about pharmacists’ clinical roles in patient care in Ghana. *Innovations in Pharmacy*, *13*(4). 10.24926/iip.v13i4.5018 PMC10256284 37305599

[CIT0015] Arastaman, G., Fidan, İ. Ö, & Fidan, T. (2018). Nitel Araştırmada Geçerlik ve Güvenirlik: Kuramsal Bir İnceleme. *Yuzunci Yil Universitesi Egitim Fakultesi Dergisi*, *15*(1), 37–75. 10.23891/efdyyu.2018.61

[CIT0016] Atac, O., Aydin, V., Karabey, S., Hayran, O., & Akici, A. (2022). Good versus poor prescribers: The comparison of prescribing competencies in primary care. *Primary Health Care Research & Development*, *23*, e22. 10.1017/S1463423622000111 35343414 PMC8991858

[CIT0017] Atikeler, E. K., Leufkens, H., & Goettsch, W. (2020). Access to medicines in Turkey: Evaluation of the process of medicines brought from abroad. *International Journal of Technology Assessment in Health Care*, *36*(6), 585–591. 10.1017/S0266462320000872 33231162

[CIT0018] Aydın, Ö. Ç., Aydın, S., & Güney, H. Z. (2020). Pharmacovigilance and radiologists: How well do they get along? *The British Journal of Radiology*, *93*(1), 20200596. 10.1259/bjr.20200596 32903029 PMC8519654

[CIT0019] Ayhan, Y. E., & Sancar, M. (2023). Interventions in internal medicine wards with scope of clinical pharmacy residency program: A retrospective study. *Ankara Universitesi Eczacilik Fakultesi Dergisi*, *47*(2), 23–23. 10.33483/jfpau.1235458

[CIT0020] Babar, Z. U. (2021). Ten recommendations to improve pharmacy practice in low and middle-income countries (LMICs). *Journal of Pharmaceutical Policy and Practice*, *14*(1), 6. 10.1186/s40545-020-00288-2 33407945 PMC7788796

[CIT0021] Babar, Z.-U.-D. (2024). A conceptual framework to build effective medicine pricing policies for low and middle-income countries (LMICs). *Research in Social and Administrative Pharmacy*. 10.1016/j.sapharm.2024.06.008 38908991

[CIT0022] Babar, Z. U., & Jamshed, S. (2008). Social pharmacy strengthening clinical pharmacy: Why pharmaceutical policy research is needed in Pakistan? *Pharmacy World & Science*, *30*(5), 617–619. 10.1007/s11096-008-9246-z 18686010

[CIT0023] Babar, Z. U. D., Kan, S. W., & Scahill, S. (2014). Interventions promoting the acceptance and uptake of generic medicines: A narrative review of the literature. *Health Policy*, *117*(3), 285–296. 10.1016/j.healthpol.2014.06.004 24973926

[CIT0024] Bayram, D., Aydin, V., Gelal, A., Aksoy, M., & Akici, A. (2021). Generic drug prescribing in primary care: A nationwide analysis. *International Journal of Clinical Practice*, *75*(8), e14284. 10.1111/ijcp.14284 33914404

[CIT0025] Bektay, M. Y., Sancar, M., Ali Jadoo, S. A., & Izzettin, F. V. (2020). Time to change to improve health: Clinical pharmacy and pharmaceutical care education in Turkey. *Journal of Ideas in Health*, *3*(1), 130–134. 10.47108/jidhealth.Vol3.Iss1.19

[CIT0026] Bektay, M. Y., Sancar, M., Okyaltirik, F., Durdu, B., & Izzettin, F. V. (2022). Investigation of drug-related problems in patients hospitalized in chest disease wards: A randomized controlled trial. *Frontiers in Pharmacology*, *13*, 1049289. 10.3389/fphar.2022.1049289 36703759 PMC9872030

[CIT0027] Bulut, S., & Bilgener, E. (2022). Evaluation of turkish pharmaceutical track and trace system (ITS): Perspective of community pharmacists. *Brazilian Journal of Pharmaceutical Sciences*, *58*. 10.1590/s2175-97902022e20407

[CIT0028] Bulut, S., Yildiz, A., & Kaya, S. (2019). Evaluation of transition to electronic prescriptions in Turkey: Perspective of family physicians. *International Journal of Health Policy and Management*, *8*(1), 40–48. 10.15171/ijhpm.2018.89 30709101 PMC6358642

[CIT0029] Buyuker, S. M., Sultana, A., Chowdhury, J. A., Chowdhury, A. A., Kabir, S., & Amran, M. S. (2023). A retrospective evaluation of self-reported adverse events following immunization with different COVID-19 vaccines in Turkiye. *Vaccines*, *11*(2). 10.3390/vaccines11020316 PMC9964774 36851193

[CIT0030] Camcioglu, Y., Sener Okur, D., Aksaray, N., Darendeliler, F., & Hasanoglu, E. (2020). Factors affecting physicians’ perception of the overuse of antibiotics. *Médecine et Maladies Infectieuses*, *50*(8), 652–657. 10.1016/j.medmal.2020.01.006 32046887

[CIT0031] Çelebi, F., Çalıkuşu, M., & Özçelikay, G. (2022). The role of community pharmacists in increasing patients’ drug compliance. *Fabad Journal of Pharmaceutical Sciences*. 10.55262/fabadeczacilik.1092310

[CIT0032] Cengiz, Z., & Ozkan, M. (2020). Development and validation of a tool to assess the rational use of drugs in Turkish adults. *Journal of Public Health*, *29*(3), 719–724. 10.1007/s10389-020-01251-w

[CIT0033] Cengiz, Z., & Ozkan, M. (2022). Applying the health belief model to the rational use of drugs for hemodialysis patients: A randomized controlled trial. *Patient Education and Counseling*, *105*(3), 679–685. 10.1016/j.pec.2021.06.024 34217550

[CIT0034] Connor, L., Dean, J., McNett, M., Tydings, D. M., Shrout, A., Gorsuch, P. F., … Gallagher-Ford, L. (2023). Evidence-based practice improves patient outcomes and healthcare system return on investment: Findings from a scoping review. *Worldviews on Evidence-Based Nursing*, *20*(1), 6–15. 10.1111/wvn.12621 36751881

[CIT0035] Demircioglu Akyilmaz, C. E., Okuyan, B., & Sancar, M. (2022). The impact of clinical pharmacist-led hypertension screening at the community pharmacy in Türkiye. *Journal of Research in Pharmacy*, *26*(6), 1900–1906. 10.29228/jrp.280

[CIT0036] Deniz-Ulutaş, E., Rasheed, M. K., Ere, R., & Gözeler, H. (2024). Lived experiences of turkish community pharmacists toward person-centric care: A qualitative analysis. *Journal of Pharmaceutical Policy and Practice*, *17*(1), 2294942. 10.1080/20523211.2023.2294942 38234994 PMC10793631

[CIT0037] The Department of Pharmacy Management at Ankara University. (2023). Retrieved February 16, 2024, from http://eczislet.pharmacy.ankara.edu.tr/en/about/.

[CIT0038] Desselle, S. P. (2005). The birth of research in social & administrative pharmacy: A sincere welcome to subscribers, readers, and authors. *Research in Social and Administrative Pharmacy*, *1*(1), 1–4. 10.1016/j.sapharm.2004.12.001 17138462

[CIT0039] Dumlu, H. I., Sancar, M., Ozdemir, A., & Okuyan, B. (2022). Impact of a clinical pharmacist-led stewardship program for the appropriate use of acid suppression therapy in older hospitalized patients: A non-randomized controlled study. *International Journal of Clinical Pharmacy*, *44*(4), 914–921. 10.1007/s11096-022-01394-8 35449351

[CIT0040] Ekmez, M., & Ekmez, F. (2022). Assessment of factors affecting attitudes and knowledge of pregnant women about COVID-19 vaccination. *Journal of Obstetrics and Gynaecology*, *42*(6), 1984–1990. 10.1080/01443615.2022.2056831 35648842

[CIT0041] Emre-Aydıngöz, S., & Lux, K. M. (2021). Türkiye’de Antibiyotik Tüketim Miktarının ve Birinci Basamak Sağlık Hizmetlerinde Antibiyotik Reçeteleme Uygulamalarının OECD Ülkeleri ile Karşılaştırmalı Olarak Değerlendirmesi. *Sağlık Bilimleri Dergisi*, *30*(1), 56–62. 10.34108/eujhs.808230

[CIT0042] Enver, C., Erturk Sengel, B., Sancar, M., Korten, V., & Okuyan, B. (2023). Medication reconciliation service in hospitalized patients with infectious diseases during coronavirus disease-2019 pandemic: An observational study. *Turkish Journal of Pharmaceutical Sciences*, *20*(4), 210–217. 10.4274/tjps.galenos.2022.08455 37605897 PMC10445224

[CIT0043] Erdoğan, ÖN, Dağıstan, ÖA, Arslan, M., Çığ, G., Sayar, A., & Erdoğan, M. S. (2021). Knowledge, attitude and practice of community pharmacy personnel about implementation of the pharmaceutical track and trace system. *Farmacia*, *69*(2), 367–374. 10.31925/farmacia.2021.2.23

[CIT0044] Ergün, Y. (2019). Farmakovijilans: Türk Mevzuatı Açısından Bir Değerlendirme. *Kahramanmaraş Sütçü İmam Üniversitesi Tıp Fakültesi Dergisi*, *14*(3), 155–161. 10.17517/ksutfd.538002

[CIT0045] Ergun, Y., Ergun, T. B., Toker, E., Unal, E., & Akben, M. (2019). Knowledge attitude and practice of Turkish health professionals towards pharmacovigilance in a university hospital. *International Health*, *11*(3), 177–184. 10.1093/inthealth/ihy073 30265317

[CIT0046] Ersoy, S., & Engin, V. S. (2019). Accessibility to healthcare and risk of polypharmacy on chronically ill patients. *Journal of the College of Physicians and Surgeons Pakistan*, *29*(6), 505–510. 10.29271/jcpsp.2019.06.505 31133145

[CIT0047] Ertuna, E., Arun, M. Z., Ay, S., Kocak, F. O. K., Gokdemir, B., & Ispirli, G. (2019). Evaluation of pharmacist interventions and commonly used medications in the geriatric ward of a teaching hospital in Turkey: A retrospective study. *Clinical Interventions in Aging*, *14*, 587–600. 10.2147/CIA.S201039 30962679 PMC6432892

[CIT0048] Fedai Kayin, I., Ciftci, H. D., Tan, B., & Akoglu, M. N. (2023). Pharmacist and child communication: A phenomenological multidisciplinary study from the perspectives of undergraduate students in pharmacy and child development. *Exploratory Research in Clinical and Social Pharmacy*, *10*, 100272. 10.1016/j.rcsop.2023.100272 37181501 PMC10172831

[CIT0049] Fernandez-Llimos, F., Desselle, S., Stewart, D., Garcia-Cardenas, V., Babar, Z. U., Bond, C., … Tonin, F. (2023). Improving the quality of publications in and advancing the paradigms of clinical and social pharmacy practice research: The Granada statements. *Research in Social and Administrative Pharmacy*, *19*(5), 830–835. 10.1016/j.sapharm.2023.01.007 36804321

[CIT0050] FIP. (2024). *Pharmacy practice research special interest group*. Retrieved February 12, 2024, from https://www.fip.org/pharmacy-practice-research

[CIT0051] Garcia-Cardenas, V., Rossing, C. V., Fernandez-Llimos, F., Schulz, M., Tsuyuki, R., Bugnon, O., … Benrimoj, S. I. (2020). Pharmacy practice research – A call to action. *Research in Social and Administrative Pharmacy*, *16*(11), 1602–1608. 10.1016/j.sapharm.2020.07.031 32919918

[CIT0052] Goker, G., Bayraktar-Ekincioglu, A., & Celebi, N. (2022). Treatment of patients with neuropathic pain and provision of drug information by clinical pharmacists. *Brazilian Journal of Pharmaceutical Sciences*, *58*. 10.1590/s2175-97902022e20390

[CIT0053] Gray, A. L., & Suleman, F. (2015). The relevance of systematic reviews on pharmaceutical policy to low- and middle-income countries. *International Journal of Clinical Pharmacy*, *37*(5), 717–725. 10.1007/s11096-015-0156-6 26177819

[CIT0054] Gül, I., Helvacıoğlu, E. T., & Saraçlı, S. (2023). Service quality, outpatient satisfaction and loyalty in community pharmacies in Turkey: A structural equation modeling approach. *Exploratory Research in Clinical and Social Pharmacy*, *12*, 100361. 10.1016/j.rcsop.2023.100361 38023638 PMC10679941

[CIT0055] Gülpınar, G., Keleş, Ş, & Yalım, N. Y. (2021). Perspectives of community pharmacists on conscientious objection to provide pharmacy services: A theory informed qualitative study. *Journal of the American Pharmacists Association*, *61*(4), 373–381. 10.1016/j.japh.2021.03.014 33895101

[CIT0056] Gülpınar, G., & Uzun, M. B. (2022). Examining community pharmacists’ intention to provide pharmacist-driven vaccination services: A structural equation modelling. *Vaccine*, *40*(1), 67–75. 10.1016/j.vaccine.2021.11.044 34844821

[CIT0057] Guven, A. T. (2023). Time to close the gap between guidelines and the reimbursement policy for diabetes treatment in Turkey. *Pharmacoeconomics*, *41*(8), 843–844. 10.1007/s40273-023-01282-7 37278901

[CIT0058] Güzeloğlu, E., & Karacı, M. (2022). Antibiotic-associated adverse drug events in hospitalized children. *Journal of Pediatric Infection*, *16*(3), 198–204. 10.5578/ced.20229714

[CIT0059] Hagens, A., Inkaya, A. C., Yildirak, K., Sancar, M., van der Schans, J., Acar Sancar, A., … Yegenoglu, S. (2021). COVID-19 vaccination scenarios: A cost-effectiveness analysis for Turkey. *Vaccines (Basel)*, *9*(4). 10.3390/vaccines9040399 PMC8073609 33919586

[CIT0060] He, W., Yao, D., Hu, Y., & Dai, H. (2018). Analysis of a pharmacist-led adverse drug event management model for pharmacovigilance in an academic medical center hospital in China. *Therapeutics and Clinical Risk Management*, *14*, 2139–2147. 10.2147/TCRM.S178297 30464487 PMC6214595

[CIT0061] Hong, Q. N., Pluye, P., Fabregues, S., Bartlett, G., Boardman, F., Cargo, M., … Vedel, I. (2018). The mixed methods appraisal tool (MMAT) version 2018 for information professionals and researchers. *Education for Information*, *34*(4), 285–291. 10.3233/EFI-180221

[CIT0062] Hussain, R., Akram, T., Hassali, M. A., Muneswarao, J., Rehman, A. U., Hashmi, F., … Babar, Z. U. (2022). Barriers and facilitators to pharmacovigilance activities in Pakistan: A healthcare professionals-based survey. *PLoS One*, *17*(7), e0271587. 10.1371/journal.pone.0271587 35905133 PMC9337632

[CIT0063] Idil, E., Aydin, A. E., Ates Bulut, E., & Isik, A. T. (2021). Rationally decreasing the number of drugs seems to be a useful therapeutic approach in older adults: 6-month follow-up study. *Archives of Gerontology and Geriatrics*, *96*, 104472. 10.1016/j.archger.2021.104472 34237523

[CIT0064] IEIS. (2023). *World and Turkish pharmaceutical market*. Retrieved February 25, 2024, from https://www.ieis.org.tr/en/world-and-turkish-pharmaceutical-market#:~:text=Turkey%20ranked%2021th%20in%202022.&text=The%20Turkish%20pharmaceutical%20market%20reached,reached%202%2C55%20billion%20units.&text=Oncology%20represented%20the%20largest%20proportion,2%25%20of%20the%20total%20market

[CIT0065] Isler, B., Keske, S., Aksoy, M., Azap, O. K., Yilmaz, M., Yavuz, S. S., … Ergonul, O. (2019). Antibiotic overconsumption and resistance in Turkey. *Clinical Microbiology and Infection*, *25*(6), 651–653. 10.1016/j.cmi.2019.02.024 30844434

[CIT0066] Joint FIP/WHO guidelines on good pharmacy practice: Standards for quality of pharmacy practice. (2011). Retrieved March 14, 2024, from https://www.who.int/docs/default-source/medicines/norms-and-standards/guidelines/distribution/trs961-annex8-fipwhoguidelinesgoodpharmacypractice.pdf

[CIT0067] Kandemir, E. A., Bayraktar-Ekincioglu, A., & Kilickap, S. (2021). Assessment of adherence to cancer-associated venous thromboembolism guideline and pharmacist's impact on anticoagulant therapy. *Supportive Care in Cancer*, *29*(3), 1699–1709. 10.1007/s00520-020-05669-6 32776163

[CIT0068] Kara, E., DemIrkan, K., & Unal, S. (2020). Knowledge and attitudes among hospital pharmacists about COVID-19. *Turkish Journal of Pharmaceutical Sciences*, *17*(3), 242–248. 10.4274/tjps.galenos.2020.72325 32636699 PMC7336039

[CIT0069] Kara, E., Kelleci Cakir, B., Sancar, M., & Demirkan, K. (2021). Impact of clinical pharmacist-led interventions in Turkey. *Turkish Journal of Pharmaceutical Sciences*, *18*(4), 517–526. 10.4274/tjps.galenos.2020.66735 34496559 PMC8430404

[CIT0070] Kara, E., Metan, G., Bayraktar-Ekincioglu, A., Gulmez, D., Arikan-Akdagli, S., Demirkazik, F., … Uzun, O. (2021). Implementation of pharmacist-driven antifungal stewardship program in a tertiary care hospital. *Antimicrobial Agents and Chemotherapy*, *65*(9), e00629–e00621. 10.1128/AAC.00629-21 34152808 PMC8370214

[CIT0071] Kara, E., Okuyan, B., Demirkan, K., & Sancar, M. (2021). A question awaiting answer during the development and implementation stage of clinical pharmacy services in Turkey: Who is clinical pharmacist? *Journal of Literature Pharmacy Sciences*, *10*(1), 109–118. 10.5336/pharmsci.2020-76878

[CIT0072] Khan, Z., Karatas, Y., & Hamid, S. M. (2023). Evaluation of health care professionals’ knowledge, attitudes, practices and barriers to pharmacovigilance and adverse drug reaction reporting: A cross-sectional multicentral study. *PLoS One*, *18*(5), e0285811. 10.1371/journal.pone.0285811 37224133 PMC10208525

[CIT0073] Kirmizi Sonmez, N. I., Aydin, V., Atac, O., & Akici, A. (2023). Association of arbitrary prescribing behavior to costly drug expenditures: A pharmacoeconomic study in primary care. *Postgraduate Medical Journal*, *100*(1179), 36–41. 10.1093/postmj/qgad092 37827534

[CIT0074] Kıran, Ş, & Akbolat, M. (2021). Availability of health resources: A comparison of Turkey and selected OECD countries. *Hacettepe Journal of Health Administration*, *24*(3), 603–618.

[CIT0075] Kockaya, G., Atalay, S., Oguzhan, G., Kurnaz, M., Okcun, S., Sar Gedik, C., … Sencan, N. (2021). Analysis of patient access to orphan drugs in Turkey. *Orphanet Journal of Rare Diseases*, *16*(1), 68. 10.1186/s13023-021-01718-3 33549137 PMC7868010

[CIT0076] Kockaya, G., Oguzhan, G., & Calskan, Z. (2020). Changes in catastrophic health expenditures depending on health policies in Turkey. *Frontiers in Public Health*, *8*, 614449. 10.3389/fpubh.2020.614449 33490026 PMC7817945

[CIT0077] Kose, I., Rayner, J., Birinci, S., Ulgu, M. M., Yilmaz, I., Guner, S., … Mo, H. T. (2020). Adoption rates of electronic health records in Turkish hospitals and the relation with hospital sizes. *BMC Health Services Research*, *20*(1), 967. 10.1186/s12913-020-05767-5 33087106 PMC7580017

[CIT0078] Koshman, S. L., & Blais, J. (2011). What is pharmacy research? *The Canadian Journal of Hospital Pharmacy*, *64*(2), 154–155. 10.4212/cjhp.v64i2.1002 22479047 PMC3093426

[CIT0079] Küçükali, H., Hayran, O., Duman, E., Karaca, A., Kirikçi, A., & Çiftçi, O. (2021). Anxiety levels of community pharmacists during the COVID-19 pandemic: A cross-sectional study in Istanbul. *Hacettepe University Journal of the Faculty of Pharmacy*. 10.52794/hujpharm.988353

[CIT0080] Kuerec, A. H., Bebitoğlu, B. T., Oğuz, E., Mesci, B., & Bulut, N. (2023). Evaluation of inappropriate drug use in geriatric patients using the TIME-to-STOP/TIME-to-START criteria in a tertiary hospital. *Journal of Research in Pharmacy*, *27*(2), 687–695. 10.29228/jrp.350

[CIT0081] Kumar, M. (2023). Emerging digital technologies for pharmaceutical drug traceability. *Universal Journal of Pharmacy and Pharmacology*, *2*(1), 34–40. 10.31586/ujpp.2023.794

[CIT0082] Malik, I., Atif, M., Scahill, S. L., & Babar, Z.-U.-D. (2020). Pharmacy practice and policy research in Pakistan: A review of literature between 2014 and 2019. In Z.-U.-D. Babar (Ed.), *Global pharmaceutical policy* (pp. 139–175). Springer Nature.

[CIT0083] Mashaki Ceyhan, E., Walker, S., & Salek, S. (2020). Patients’ perspectives of the pharmaceutical regulatory and reimbursement systems in Istanbul, Turkey. *Therapeutic Innovation & Regulatory Science*, *54*(5), 1086–1096. 10.1007/s43441-020-00124-4 32030691

[CIT0084] Ministry of Health Malaysia. (2024). *Pharmacy research priorities in Malaysia*. Pharmaceutical Services Programme, Ministry of Health Malaysia. Retrieved April 18, 2024, from https://pharmacy.moh.gov.my/en/documents/pharmacy-research-priorities-malaysia-second-edition.html

[CIT0085] Ministry of Health of the Republic of Türkiye. (2019). *Kamu sağlık tesisleri ruhsatlandırma yönetmeliği*. Retrieved February 25, 2024, from https://www.resmigazete.gov.tr/eskiler/2019/09/20190920-2.htm

[CIT0086] Ministry of Health of the Republic of Türkiye. (2023). *Strategic plan: 2019–*2023. Retrieved February 25, 2024, from https://sgb.saglik.gov.tr/Eklenti/37312/0/stratejik-plan-2020-ingilizcepdf.pdf?_tag1=5326746E973C7229E9E9210476EA794341993162

[CIT0087] Obaid, D., El-Dahiyat, F., & Babar, Z. U. (2022). Pharmacy practice and clinical pharmacy research in the Middle East: A scoping review of studies from Bahrain, Iraq, Jordan, Kuwait, Lebanon, Oman, Palestine, Qatar, Saudi Arabia, Syria, United Arab Emirates, and Yemen. *Journal of Pharmaceutical Policy and Practice*, *15*(1), 40. 10.1186/s40545-022-00434-y 35676727 PMC9175494

[CIT0088] OECD. (2018). *Stemming the superbug tide: Just a few dollars more*. Retrieved April 27, 2024, from https://www.oecd.org/health/stemming-the-superbug-tide-9789264307599-en.htm

[CIT0089] OECD. (2023). Health at a glance 2023 country note: Türkiye. Retrieved February 25, 2024, from https://www.oecd.org/turkiye/health-at-a-glance-T%C3%BCrkiye-EN.pdf

[CIT0090] Okem, Z. G., & Cakar, M. (2015). What have health care reforms achieved in Turkey? An appraisal of the “Health Transformation Programme”. *Health Policy*, *119*(9), 1153–1163. 10.1016/j.healthpol.2015.06.003 26183890

[CIT0091] Oksuz, E., Malhan, S., Gonen, M. S., Kutlubay, Z., Keskindemirci, Y., Jarrett, J., … Tabak, F. (2021). Cost-effectiveness analysis of remdesivir treatment in COVID-19 patients requiring low-flow oxygen therapy: Payer perspective in Turkey. *Advances in Therapy*, *38*(9), 4935–4948. 10.1007/s12325-021-01874-9 34379304 PMC8355577

[CIT0092] Okuyan, B., Balta, E., Ozcan, V., Durak Albayrak, O., Turker, M., & Sancar, M. (2021). Turkish community pharmacists’ behavioral determinants in provision of pharmaceutical care to elderly patients. *International Journal of Clinical Pharmacy*, *43*(4), 1024–1035. 10.1007/s11096-020-01211-0 33411182

[CIT0093] Okuyan, B., Bektay, M. Y., Kingir, Z. B., Save, D., & Sancar, M. (2021). Community pharmacy cognitive services during the COVID-19 pandemic: A descriptive study of practices, precautions taken, perceived enablers and barriers and burnout. *International Journal of Clinical Practice*, *75*(12), e14834. 10.1111/ijcp.14834 34510660 PMC8646293

[CIT0094] Okuyan, B., Ozcan, V., Balta, E., Durak-Albayrak, O., Turker, M., Sancar, M., … Ozcebe, H. (2021). The impact of community pharmacists on older adults in Turkey. *Journal of the American Pharmacists Association*, *61*(6), e83–e92. 10.1016/j.japh.2021.06.009 34238671

[CIT0095] Oncu, S., Bayram, D., Aydin, V., Isli, F., Aksoy, M., Akici, A., … Gelal, A. (2021). Knowledge, opinions and attitudes of primary care physicians about generic drugs: A cross-sectional study. *Family Practice*, *38*(3), 272–279. 10.1093/fampra/cmaa138 33340330

[CIT0096] Ozcebe, H., Erguder, T., Balcilar, M., Ursu, P., Reeves, A., Stuckler, D., … Mauer-Stender, K. (2018). The perspectives of politicians on tobacco control in Turkey. *European Journal of Public Health*, *28*(suppl_2), 17–21. 10.1093/eurpub/cky152 30371833 PMC6204546

[CIT0097] Ozdamar, I., & Ozdamar, E. N. (2021). Drug utilization pattern and rational drug use at orthopedics and traumatology outpatient clinics: A cross-sectional study. *Joint Diseases and Related Surgery*, *32*(3), 759–766. 10.52312/jdrs.2021.211 34842110 PMC8650645

[CIT0098] Ozdemir, N., Kara, E., Bayraktar-Ekincioglu, A., Buyukcam, A., Celiker, A., Demirkan, K., … Kara, A. (2022). Knowledge, attitudes, and practices regarding vaccination among community pharmacists. *Primary Health Care Research & Development*, *23*, e38. 10.1017/S1463423622000330 35866296 PMC9309755

[CIT0099] Ozturk, S., Basar, D., Ozen, I. C., & Ciftci, A. O. (2019). Socio-economic and behavioral determinants of prescription and non-prescription medicine use: The case of Turkey. *DARU Journal of Pharmaceutical Sciences*, *27*(2), 735–742. 10.1007/s40199-019-00311-1 31732873 PMC6895375

[CIT0100] Ozturk, O., Sunter, A. T., Unal, M., Selcuk, M. Y., & Oruc, M. A. (2021). Evaluation of painkillers according to the principles of rational drug use in patients registered to a family medicine unit. *International Journal of Clinical Practice*, *75*(5), e14018. 10.1111/ijcp.14018 33428818

[CIT0101] Parmaksız, K., Pisani, E., & Kok, M. O. (2020). What makes a national pharmaceutical track and trace system succeed? Lessons from Turkey. *Global Health: Science and Practice*, *8*(3), 431–441. 10.9745/GHSP-D-20-00084 33008856 PMC7541108

[CIT0102] Pehlivanlı, A., Selçuk, A., Eyüpoğlu, Ş, Ertürk, Ş, & Özçelikay, A. T. (2022). Potentially inappropriate medication use in older adults with chronic kidney disease. *Turkish Journal of Pharmaceutical Sciences*, *19*(3), 305–313. 10.4274/tjps.galenos.2021.94556 35775387 PMC9254095

[CIT0103] Pehlivanli, A., Eyupoglu, S., Basgut, B., Erturk, S., & Ozcelikay, A. T. (2023). Impact of a multidisciplinary approach involving clinical pharmacist on resolving drug related problems in chronic kidney patients: A prospective interventional study. *BMC Nephrology*, *24*(1), 149. 10.1186/s12882-023-03210-5 37237342 PMC10224574

[CIT0104] Phougat, P., Beniwal, M., Kapoor, G., Aggarwal, N., Kumari, A., Sharma, R., … Kamal, M. A. (2024). Role and responsibilities of various stakeholders in pharmacovigilance (PV). *Current Drug Safety*. 10.2174/0115748863277574240125045459 38318830

[CIT0105] Roy, V., & Rana, P. (2018). Prescribing generics: All in a name. *Indian Journal of Medical Research*, *147*(55), 442–444. 10.4103/ijmr.IJMR_1940_17 30082567 PMC6094511

[CIT0106] Ryan, M., Romanelli, F., Smith, K., & Johnson, M. M. S. (2003). Identifying and teaching generation X pharmacy students. *American Journal of Pharmaceutical Education*, *67*(2), 42. 10.1016/S0002-9459(24)00735-6

[CIT0107] Şahin, Y., Nuhoğlu, Ç, Okuyan, B., & Sancar, M. (2022). Assessment of drug-related problems in pediatric inpatients by clinical pharmacist-led medication review: An observational study. *Journal of Research in Pharmacy*, *26*(4), 1007–1015. 10.29228/jrp.198

[CIT0108] Savas, M., Bayraktar-Ekincioglu, A., & Celebi, N. (2021). An evaluation of cancer patients’ opinions about use of opioid analgesics and the role of clinical pharmacist in patient education in Turkey. *International Journal of Clinical Pharmacy*, *43*(2), 375–382. 10.1007/s11096-020-01098-x 32740850

[CIT0109] Schultz, B. G., Tilton, J., Jun, J., Scott-Horton, T., Quach, D., & Touchette, D. R. (2021). Cost-effectiveness analysis of a pharmacist-led medication therapy management program: Hypertension management. *Value in Health*, *24*(4), 522–529. 10.1016/j.jval.2020.10.008 33840430

[CIT0110] Sumbul-Sekerci, B., Bilgic, B., Pasin, O., Emre, M., & Hanagasi, H. A. (2022). Anticholinergic burden, polypharmacy, and cognition in Parkinson's disease patients with mild cognitive impairment: A cross-sectional observational study. *Dementia and Geriatric Cognitive Disorders*, *51*(5), 386–395. 10.1159/000526863 36273437 PMC9909708

[CIT0111] Tacconelli, E. (2010). Systematic reviews: CRD's guidance for undertaking reviews in health care. *The Lancet Infectious Diseases*, *10*(4), 226. 10.1016/S1473-3099(10)70065-7

[CIT0112] Tarhan, N., & Arslan, M. (2023). Psychometric assessment of pharmacists’ counseling in dementia. *Patient Education and Counseling*, *115*, 107903. 10.1016/j.pec.2023.107903 37506523

[CIT0113] TC. Sağlık Bakanlığı. Sağlıkta İnsan Kaynakları 2023 Vizyonu. (2011). Retrieved April 28, 2024, from https://shgmsigpdb.saglik.gov.tr/Eklenti/39431/0/saglikta-insan-kaynaklari-2023-vizyonupdf.pdf

[CIT0114] Tengilimoğlu, D., Tekin, PŞ, Zekioğlu, A., & Kılıç, T. D. (2020). Consumer awareness, attitude, and behavior related to the rational use of medicines in a developing country context: The case of Turkey. *Open Access Macedonian Journal of Medical Sciences*, *8*(E), 162–171. 10.3889/oamjms.2020.3912

[CIT0115] Tengiz, I., Atila, D., & Ercan, E. (2023). Market distributions and pricing/reimbursement policies of antihypertensive drugs in Turkey. *Turk Kardiyoloji Dernegi Arsivi-Archives of the Turkish Society of Cardiology*, *51*(5), 299–303. 10.5543/tkda.2023.84039 37450453

[CIT0116] TÜİK. (2022a). *Death and causes of death statistics*. https://data.tuik.gov.tr/Bulten/Index?p=Death-and-Causes-of-Death-Statistics-2022-49679&dil=2

[CIT0117] TÜİK. (2022b). *Health expenditure statistics*. Retrieved February 25, 2024, from https://data.tuik.gov.tr/Bulten/Index?p=49676&dil=2

[CIT0118] TÜİK. (2024). *İstatistik Veri Portalı*. https://data.tuik.gov.tr/Kategori/GetKategori?p=Nufus-ve-Demografi-109

[CIT0119] Tonin, F. S., Aznar-Lou, I., Pontinha, V. M., Pontarolo, R., & Fernandez-Llimos, F. (2021). Principles of pharmacoeconomic analysis: The case of pharmacist-led interventions. *Pharmacy Practice*, *19*(1), 2302. 10.18549/PharmPract.2021.1.2302 33727994 PMC7939117

[CIT0120] Topcuoglu, M. A., & Arsava, E. M. (2021). Time in therapeutic range among warfarin users in Turkey: Are there enough data to set definitive criteria for reimbursement? *Turk Kardiyoloji Dernegi Arsivi-Archives of the Turkish Society of Cardiology*, *49*(4), 254–256. 10.5543/tkda.2021.94055 34106058

[CIT0121] Tugay, D., Top, M., Aydin, O., Bavbek, S., Damadoglu, E., Oner Erkekol, F., … Abraham, I. (2023). Real-world patient-level cost-effectiveness analysis of omalizumab in patients with severe allergic asthma treated in four major medical centers in Turkey. *Journal of Medical Economics*, *26*(1), 720–730. 10.1080/13696998.2023.2209417 37129881

[CIT0122] Umar, R. M., Apikoglu-Rabus, S., & Yumuk, P. F. (2020). Significance of a clinical pharmacist-led comprehensive medication management program for hospitalized oncology patients. *International Journal of Clinical Pharmacy*, *42*(2), 652–661. 10.1007/s11096-020-00992-8 32078106

[CIT0123] Uzun, G. D., Sancar, M., & Okuyan, B. (2019). Evaluation of knowledge and attitude of pharmacist and pharmacy technicians on emergency contraception method in Istanbul, Turkey: A simulated patient study. *Journal of Research in Pharmacy*, *23*(3), 395–402. 10.12991/jrp.2019.147

[CIT0124] Vogler, S., Zimmermann, N., Haasis, M. A., Knoll, V., Espin, J., Mantel-Teeuwisse, A. K., … Babar, Z. U. (2024). Innovations in pharmaceutical policies and learnings for sustainable access to affordable medicines. *Journal of Pharmaceutical Policy and Practice*, *17*(Suppl 1), 2335492. 10.1080/20523211.2024.2335492 38757122 PMC11095271

[CIT0125] Westerling, R., Daryani, A., Gershuni, O., Czabanowska, K., Brand, H., Erdsiek, F., … Brzoska, P. (2020). Promoting rational antibiotic use in Turkey and among Turkish migrants in Europe – Implications of a qualitative study in four countries. *Globalization and Health*, *16*(1), 108. 10.1186/s12992-020-00637-5 33176820 PMC7656668

[CIT0126] The World Bank. (2020). *Current health expenditure per capita- Türkiye*. Retrieved February 25, 2024, from https://data.worldbank.org/indicator/SH.XPD.CHEX.PC.CD?locations=TR

[CIT0127] Yazicioglu, B., & Yardan, E. D. (2021). Rational drug use in elderly patients in a primary care center. *Journal of the Pakistan Medical Association*, *71*(5), 1353–1356. 10.47391/JPMA.909 34091614

[CIT0128] Yildirim, F., Buyukkayaci Duman, N., Sahin, E., & Vural, G. (2023). The effect of kangaroo care on paternal attachment: A randomized controlled study. *Advances in Neonatal Care*, *23*(6), 596–601. 10.1097/ANC.0000000000001100 37884013

[CIT0129] Yildirim, Z., & Kasikci, M. (2023). The effect of education on self-care agency and rational drug use of patients with COPD. *Patient Education and Counseling*, *114*, 107804. 10.1016/j.pec.2023.107804 37257261

[CIT0130] Yilmaz, Z., & Bulut, K. (2023). Necessity and impact of patient education driven by pharmacist on the knowledge level of diabetic patients. *Journal of Research in Pharmacy*, *27*(5), 1911–1923. 10.29228/jrp.473

[CIT0131] Yilmaz, Z. K., & Sencan, N. (2021). An examination of the factors affecting community pharmacists’ knowledge, attitudes, and impressions about the COVID-19 pandemic. *Turkish Journal of Pharmaceutical Sciences*, *18*(5), 530–540. 10.4274/tjps.galenos.2020.01212 34708643 PMC8562124

[CIT0132] Yılmaz, Z., Sancar, M., Okuyan, B., Yeşildağ, O., & İzzettin, F. V. (2020). Impact of verbal and web-based patient education programs driven by clinical pharmacist on the adherence and illness perception of hypertensive patients. *Indian Journal of Pharmaceutical Education and Research*, *54*(3s), s695–s704. 10.5530/ijper.54.3s.170

[CIT0133] Yumrukaya, L., Postma, M. J., Sözen-Şahne, B., & Yeğenoğlu, S. (2022). Recommendations on pharmacoeconomic guidelines for Turkey considering reference countries: A scoping review. *Health Policy and Technology*, *11*(4). 10.1016/j.hlpt.2022.100682

