# Supplementary material for: Pharmacy practice and policy research in Türkiye: a systematic review of literature
Source: J Pharm Policy Pract. 2024 Aug 12;17(1):2385939. doi: 10.1080/20523211.2024.2385939 (PMC11321099; doi:10.1080/20523211.2024.2385939)
Supplement: Figure.pptx [file JPPP_A_2385939_SM9048.pptx]

## Slide 1
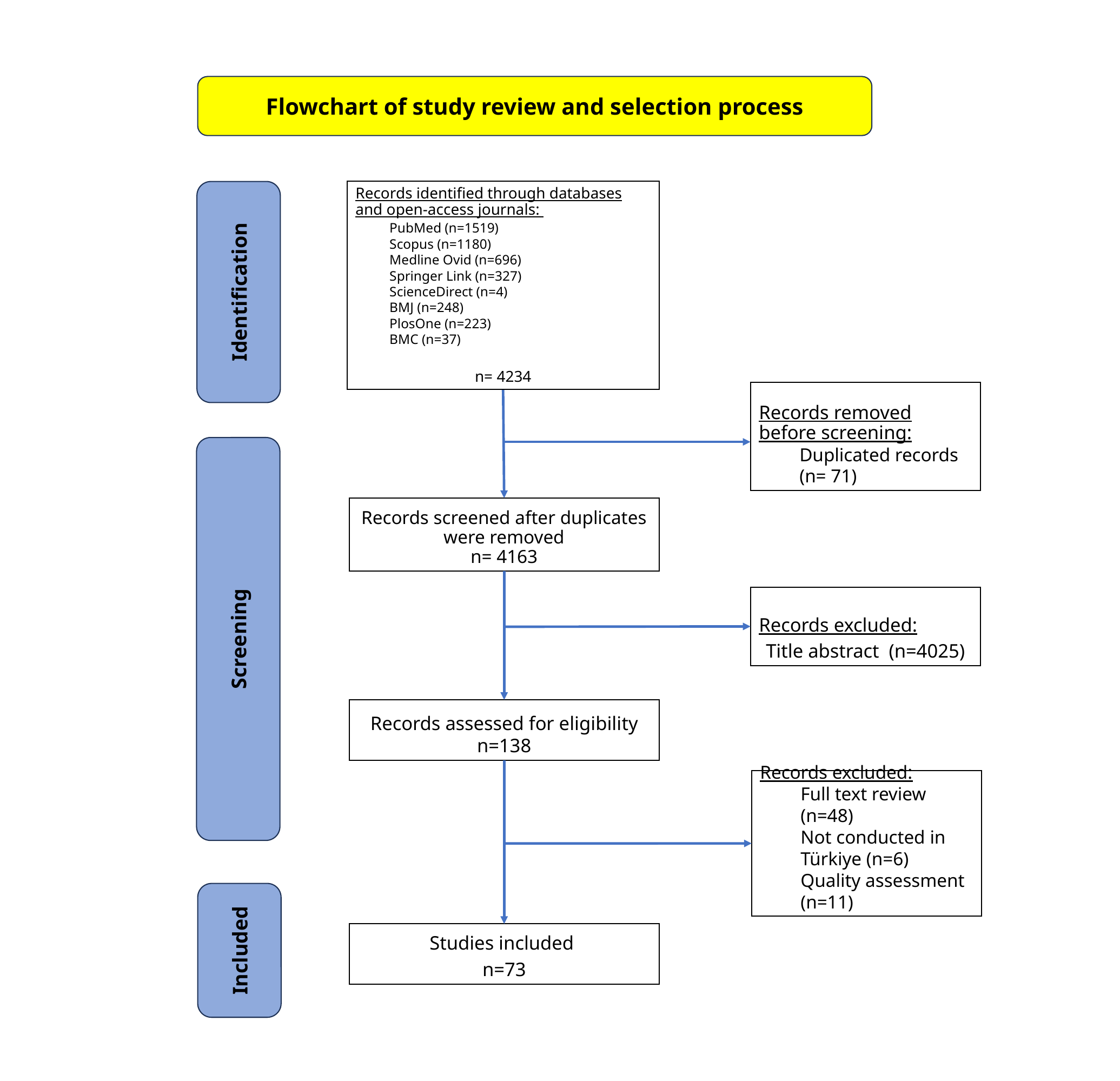

Flowchart of study review and selection process
Records identified through databases and open-access journals:
PubMed (n=1519)
Scopus (n=1180)
Medline Ovid (n=696)
Springer Link (n=327)
ScienceDirect (n=4)
BMJ (n=248)
PlosOne (n=223)
BMC (n=37)
n= 4234
Identification
Records removed before screening:
Duplicated records (n= 71)
Records screened after duplicates were removed
n= 4163
Records excluded:
Title abstract (n=4025)
Screening
Records assessed for eligibility
n=138
Records excluded:
Full text review (n=48)
Not conducted in Türkiye (n=6)
Quality assessment (n=11)
Included
Studies included
n=73
